# French-Canadian Translation and Cultural Adaptation of the Clinical Opiate Withdrawal Scale: The COWS-FC

**DOI:** 10.1177/07067437221087066

**Published:** 2022-03-15

**Authors:** Alice Bruneau, Clarice Poirier, Mélanie Bérubé, Aline Boulanger, Céline Gélinas, Line Guénette, Anaïs Lacasse, David Lussier, Yannick Tousignant-Laflamme, M. Gabrielle Pagé, Marc O. Martel, On behalf of the Quebec Pain Research Network (QPRN) Strategic Initiative on Opioids

**Affiliations:** 1 Faculty of Medicine, 5620 McGill University, Montreal, Quebec, Canada; 2 Faculty of Medicine, Université de Montréal, Montreal, Quebec, Canada; 3 Faculty of Nursing, Université Laval, Québec City, Quebec, Canada; 4 177460 Centre de recherche du Centre hospitalier universitaire de Québec, Population Health and Optimal Health Practices Research Unit, Québec City, Quebec, Canada; 5 Department of Anesthesiology and Pain Medicine, Faculty of Medicine, Université de Montréal, Montreal, Quebec, Canada; 6 Pain Clinic, Centre hospitalier de l’Université de Montréal, Montreal, Quebec, Canada; 7 Ingram School of Nursing, 5620 McGill University, Montreal, Quebec, Canada; 8 Faculty of Pharmacy, 4440 Laval University, Quebec City, Quebec, Canada; 9 Department of Health Sciences, 7001 Université du Québec en Abitibi-Témiscamingue, Rouyn-Noranda, Quebec, Canada; 10 Centre de recherche, l’Institut universitaire de gériatrie de Montréal du CIUSSS du Centre-Sud-de-l’Ile-de Montréal, Montreal, Quebec, Canada; 11 Département de médecine, Faculté de médecine, Université de Montréal, Montreal, Quebec, Canada; 12 School of Rehabilitation, Faculty of Medicine and Health Sciences, 7321 Université de Sherbrooke, Sherbrooke, Quebec, Canada; 13 Centre de recherche, Centre hospitalier de l’ 5622 Université de Montréal (CRCHUM), Montreal, Quebec, Canada; 14 Faculty of Dentistry & Department of Anesthesiology, 5620 McGill University, Montreal, Quebec, Canada

**Keywords:** opioids, pain, withdrawal, opioid use disorder

## Abstract

The assessment of opioid withdrawal symptoms is common in both clinical and research settings. The Clinical Opiate Withdrawal Scale (COWS) is among the most frequently used instruments for the assessment of signs and symptoms associated with opioid withdrawal. The COWS is a validated, clinician-administered instrument initially developed and validated for English-speaking populations. To date, however, the COWS has yet to be linguistically and culturally adapted for French-Canadian populations.

**Objective:**

The main objective of the present study was to develop a French-Canadian translation and adaptation of the COWS (i.e., the COWS-FC) for the assessment of opioid withdrawal symptoms in clinical and research settings.

**Methods:**

The French-Canadian translation and cultural adaptation of the COWS was performed following guidelines for the translation and cross-cultural adaptation of self-report measures. The steps consisted of (1) initial translation from English to French, (2) synthesis of the translation, (3) back-translation from French to English, (4) expert committee meeting, (5) test of the prefinal version among healthcare professionals and (6) review of final version by the expert committee. The expert committee considered four major areas where the French-Canadian version should achieve equivalence with the original English-version of the COWS. These areas were (1) semantic equivalence; (2) idiomatic equivalence; (3) experiential equivalence and (4) conceptual equivalence.

**Results:**

Rigorous steps based on the guidelines for the translation and cultural adaptation of assessment tools were followed, which led to a semantically equivalent version of the COWS. After a pretest among healthcare professionals, members from the expert committee agreed upon slight modifications to the French-Canadian version of the COWS to yield a final COWS-FC version.

**Conclusions:**

A French-Canadian translation and adaptation of the COWS (i.e., the COWS-FC) was developed. The COWS-FC could be used for the assessment of opioid withdrawal symptoms in clinical and research settings.

## Abrégé

L’évaluation des symptômes de sevrage des opioïdes est commune tant en milieu clinique qu’en recherche. L’Échelle de symptômes de sevrage des opioïdes (COWS) est parmi les instruments les plus fréquemment utilisés pour évaluer les signes et les symptômes associés au sevrage des opioïdes. L'échelle COWS est un instrument validé administré par un clinicien, initialement mis au point et validé pour des populations anglophones. À ce jour, toutefois, L'échelle COWS doit encore être adaptée sur le plan linguistique et culturel pour les populations canadiennes-françaises.

**Objectif:**

Le principal objectif de la présente étude était d’élaborer une traduction canadienne-française et une adaptation de l'échelle COWS pour l’évaluation des symptômes de sevrage des opioïdes en milieu clinique et de recherche.

**Méthodes:**

La traduction canadienne-française et l’adaptation culturelle de l'échelle COWS ont été menées conformément aux lignes directrices de la traduction et de l’adaptation transculturelle des mesures d’auto-évaluation. Les étapes consistaient en (1) la traduction initiale de l’anglais au français, (2) la synthèse de la traduction, (3) une traduction à rebours du français à l’anglais, (4) une réunion du comité d’experts, (5) essai de la version pré-finale chez les professionnels des soins de santé, et (6) révision de la version finale par le comité d’experts. Le comité d’experts déterminait quatre domaines majeurs où la version canadienne-française devait atteindre l’équivalence avec la version originale de l'échelle COWS. Ces domaines étaient (1) l’équivalence sémantique; (2) l’équivalence idiomatique; (3) l’équivalence expérientielle; et (4) l’équivalence conceptuelle.

**Résultats:**

Des mesures rigoureuses basées sur les lignes directrices de la traduction et de l’adaptation culturelle des instruments d’évaluation ont été suivies, ce qui a entraîné une version sémantiquement équivalente de l'échelle COWS. Après un test préliminaire chez des professionnels de la santé, les membres du comité d’experts ont convenu de légères modifications à la version canadienne-française de l'échelle COWS pour livrer la version finale.

**Conclusions:**

Une version canadienne-française et une adaptation de l'échelle COWS a été développée. L'échelle COWS-CF peut servir à évaluer les symptômes de sevrage des opioïdes en milieu clinique et recherche.

## Introduction

Opioids refer to a class of natural, synthetic or semi-synthetic drugs that bind to opioid receptors. Opioids are commonly used for medical reasons (e.g., pain relief), but they are also used illicitly for recreational reasons. Whether used for medical or recreational reasons, it is well recognized that the prolonged use of opioids may lead to physical dependence due to opioid-induced neurobiological adaptations in the central nervous system.^1–5^ Signs of physical dependence on opioids are typically manifested by opioid withdrawal symptoms following cessation of opioids, decreases in opioid blood levels or administration of an opioid antagonist.^2,3,6–8^ Opioid withdrawal may be experienced with varying intensity and may include signs and symptoms such as tremors, sweating, yawning, piloerection, lacrimation, rhinorrhea, nausea, achiness, restlessness and dysphoria.^2,3,6–8^ Opioid withdrawal symptoms are mainly observed among illicit opioid users presenting with opioid use disorder (OUD),^7–11^ but transient opioid withdrawal symptoms may also be experienced towards the end of dosing intervals among patients prescribed daily opioid therapy,^2,12–18^ or when patients undergo opioid dose reduction.^19–23^

The assessment of opioid withdrawal symptoms is common in both clinical and research settings. In the clinical settings, the assessment of opioid withdrawal symptoms is routinely performed for diagnostic or treatment planning purposes^3,7,24,25^ as well as in the context of buprenorphine–naloxone induction.^26–30^ It is also important to assess opioid withdrawal symptoms because they lead to psychological distress and contribute to decreased quality of life.^18,31–34^ In research settings, opioid withdrawal symptoms are commonly assessed in studies testing the effectiveness of pharmacological^26,35,36^ and non-pharmacological^37–39^ interventions for individuals with OUD.

A number of instruments have been developed and validated for the assessment of opioid withdrawal symptoms, including the Opiate Withdrawal Scale (OWS^40^), the Objective Opiate Withdrawal Scale^41^, the Subjective Opiate Withdrawal Scale^42^, the Short Opiate Withdrawal Scale (SOWS-G^43^), the Subjective Opiate Withdrawal Questionnaire (SOWQ^44^), the Adjective Rating Scale for Withdrawal (ARSW^45^) and the Clinical Opiate Withdrawal Scale (COWS^46^). To date, however, none of these instruments have been linguistically or culturally adapted for French-Canadian populations. Among these instruments, the COWS is one of the most commonly used. The COWS is a validated, clinician-administered instrument that can be used to quantify the frequency and severity of eleven common signs and symptoms associated with opioid withdrawal. It was developed for American English-speaking populations in the early 2000s by Wesson and Ling^46^ and later validated by Tompkins and colleagues.^9^ Because of its good psychometric properties, clinical utility and ease of application, the COWS has become widely used for the assessment of opioid withdrawal symptoms, both in clinical and research settings.^47–52^

The main objective of the present study was to develop a French-Canadian translation and adaptation of the COWS (i.e., the COWS-FC) that can be used for the assessment of opioid withdrawal symptoms in clinical and research settings. As recommended,^53^ linguistic translations of instruments also need to be culturally adapted to the target population. Given that up to seven million of Canadians report French as their first official spoken language,^54^ there is a need to develop a culturally adapted version of the COWS that could be used by French Canadians in the province of Quebec as well as in other Canadian provinces and territories.

## Methods

### Original Instrument: The COWS

The COWS was first published as a training manual for OUD treatment with buprenorphine.^46^ It was developed by Wesson and Ling in response to increased rates of opioid prescription among pain patients, as well as a gain in popularity of sublingual buprenorphine in the United States. It was designed as a clinician-administered instrument that rates 11 common opioid withdrawal signs and symptoms^46^ and took into consideration that each sign and symptom may occur along a spectrum, as reflected by the use of graded responses. The COWS consists of 11 items, including 1 subjective symptom item, 6 objective sign items and 4 items that included subjective and objective components. The final score is a sum of all items, with the following cut-off points: 5–12 = mild; 13–24 = moderate; 25–36 = moderately severe; more than 36 = severe withdrawal. The scale was validated in a sample of 46 out-of-treatment opioid-dependent volunteers.^9^ The initial validation revealed a good internal consistency, with a Cronbach α of 0.78.^9^

The COWS was specifically selected by our expert committee among the various scales for linguistic and cultural adaptation given its psychometric properties, its previous validation as an instrument for the assessment of withdrawal symptoms among various populations of opioid users,^55–62^ as well as for its ease of administration by clinicians.

### Procedures

The translation and cultural adaptation of the COWS was performed following guidelines for the translation and cross-cultural adaptation of self-report measures published by Beaton and colleagues.^53^ All the translation and cultural adaptation procedures are described below.

#### Step 1: Initial (French) translation

The first step of the translation process was the forward translation from the original language (i.e., English) to the target language (i.e., French-Canadian) by two bilingual individuals from the province of Quebec, speaking French-Canadian as their native language. These two translators had the ability to speak both languages with the facility of a native speaker. The first translator was an individual working in the field of pain and opioids (T1), and the second translator was a certified French linguist with no biomedical background and without any a priori knowledge of the concepts being measured (T2). Each individual involved in the translation process produced a Canadian French version of the COWS and a detailed report summarizing the rationale for their choices of words, as well as comments regarding potentially challenging phrases and concepts.

#### Step 2: Synthesis of the translations

A synthesis of the two translated versions (from T1 and T2) was made following the independent translations of the COWS. The two independent translators and the research coordinators met through a video conference platform to examine the results of the first step and to reconcile any discrepancies. At the end of the meeting, the team agreed upon a common French-Canadian version and provided a unified preliminary version of the COWS.

#### Step 3: Back translation

The third step consisted of a back-translation of the questionnaire (i.e., from French to English) by bilingual translators from Canada, speaking English as their native language. One of the back-translators (BT1) was an individual working in the field of pain and opioids, and the other back-translator (BT2) was a certified English linguist with no biomedical background and/or knowledge of the field. Both individuals were blinded to the original version of the COWS. As in the first step, each individual involved in the back-translation process generated an individual back-translated version of the COWS accompanied by a detailed report of their rationale for the choice of words and comments regarding potentially challenging phrases or concepts.

Following the back translation, a synthesis meeting was held between the two individuals involved in the back translation and the research coordinator to compare the two back-translated versions and to reconcile any discrepancies. At the end of the synthesis meeting, a global report was created, which included the original English version of the COWS, the two French-Canadian versions, and the reports of the forward translators, the unified T-12 version, the two English back translations, and the reports from the back translators.

#### Step 4: Expert committee

The fifth stage involved an expert committee composed of healthcare professionals (i.e., physicians, nurses and clinical psychologists), researchers working in the field of opioids and research coordinators. The global report of the preceding step (i.e., Step 3) was sent to all committee members before the meeting. A teleconference (using a web-based screen-sharing system) was then organized with the objective of generating a prefinal French-Canadian version of the COWS. As recommended by Beaton et al.,^53^ the experts considered four major areas where the French-Canadian version should achieve equivalence with the original English version of the COWS. The areas are (1) semantic equivalence (i.e., the meaning of words); (2) idiomatic equivalence (i.e., the meaning of expressions or idioms); (3) experiential equivalence (i.e., the adaptation or replacement of items that are specific to the target culture); and (4) conceptual equivalence (i.e., the definition of concepts that are common to both cultures).

#### Step 5: Test of the prefinal version

For the fifth step, the prefinal French-Canadian version of the COWS was pretested in a sample of 28 healthcare professionals working in francophone university-affiliated hospitals in the province of Quebec (see Table 1). The healthcare professionals that were recruited for the pretest were clinician working in the area of chronic pain or substance use (anesthesiologists, nurses, psychiatrists, pharmacists, family physicians). The healthcare professionals also had to be aged 18 years old or above and speak French as their native language. All procedures related to the pretest were approved by the Ethics Review Board of the Centre hospitalier de l’Université de Montréal.

**Table 1.** Characteristics of the Health Care Professionals Participating in the Study (*N* = 28).

	%
Age (years)	
18–34	22
35–44	41
45–54	15
55–65	22
Gender	
Women	78
Men	22
Ethnicity	
Caucasian (White)	93
Other	7
Occupation	
Nurse clinician	30
Nurse technician	18
Nurse auxiliary	4
Pharmacist	11
General practitioner	15
Anaesthesiologist	22
Years of experience in their occupation	
≤5	11
6–10	19
11–15	11
16–20	26
21–25	11
26–30	7
>30	15
Years of experience with opioid users	
≤5	23
6–10	12
11–15	30
16–20	15
21–25	4
26–30	4
>30	12

Between October 2019 and December 2019, the convenience sample of healthcare professionals was contacted through institutional emails and invited to participate in the pretesting step, which took place through a web-based questionnaire. After providing consent, healthcare professionals were asked to read all instructions and items from the prefinal French-Canadian version of the COWS. They were then asked to provide open-ended comments regarding the clarity of each item. After providing comments on the prefinal French-Canadian version of the COWS, participants (i.e., healthcare professionals) were asked to provide sociodemographic information (i.e., age, gender, ethnicity) as well as information about their occupation and years of experience working with opioid users. The pretest took approximately 15 minutes in total, and no financial compensation was offered.

#### Step 6: Final version

During the final step, members from the expert committee agreed upon slight modifications to the French-Canadian version of the COWS after reviewing the results obtained in the pretest (see Table 2). The final version of the COWS-FC is presented in Online Appendix 1.

**Table 2.** Overview of Results from the Pretest and Subsequent Changes Made to Yield the COWS-FC.

Items of the Original COWS	Original Text	Penultimate French-Canadian Version	Comments from Clinicians Participating in the Survey	COWS-FC
Title	Clinical Opiate Withdrawal Scale	Échelle du sevrage clinique des opioïdes	N/A	Échelle du sevrage clinique des opioïdes
Instructions	For each item, circle the number that best describes the patient's signs or symptom. Rate on just the apparent relationship to opiate withdrawal. For example, if heart rate is increased because the patient was jogging just prior to assessment, the increase pulse rate would not add to the score.	Pour chaque item, veuillez encercler le nombre correspondant le mieux aux signes et symptômes du patient. Effectuez l’évaluation en vous basant uniquement sur les symptômes spécifiques au sevrage des opioïdes. Par exemple, si la fréquence cardiaque augmente parce que le patient est allé courir juste avant l’évaluation, vous ne devez pas tenir compte de l’augmentation de la fréquence cardiaque dans le score.	Clear, simple or no comments (*n* = 18) The evaluation should take in consideration the patient history (*n* = 1) Expressed concerns about the consideration of the pulse rate measure if patient exercised (*n* = 2) Suggested using the word “chiffre” instead of the word “nombre” (*n* = 2) Text could be shorter (*n* = 2) Could clarify that we are observing signs and symptoms since this is a clinician-observed scale (*n* = 2)	Pour chaque item, veuillez encercler le nombre correspondant le mieux aux signes et symptômes du patient. Effectuez l’évaluation en vous basant sur les symptômes spécifiques au sevrage des opioïdes. Par exemple, si la fréquence cardiaque augmente parce que le patient est allé courir juste avant l’évaluation, vous ne devez pas tenir compte de l’augmentation de la fréquence cardiaque dans le score.
Resting pulse rate	Resting pulse rate (_____ beats/min): *Measured after patient is sitting or lying for one minute.* 0 pulse rate 80 or below 1 pulse rate 81–100 2 pulse rate 101–120 4 pulse rate greater than 120	Fréquence cardiaque au repos: mesurée après une minute assis ou couché _______ /min. 0 pouls à 80 ou moins 1 pouls entre 81–100 2 pouls entre 101–120 4 pouls à plus de 120	Clear, simple or no comments (*n* = 21) Could be pertinent to indicate the resting pulse rate after 2–10 min sitting down (*n* = 3) Pulse rate is usually measured over 15 s (*n* = 1) Suggested indicating the numbers are “points” (*n* = 1) Suggested rewording “Notez la fréquence cardiaque au repos” (*n* = 1) Certain medications can have an impact on the pulse rate (*n* = 1) Adding « pouls » at each answer is redundant (*n* = 1)	1. Fréquence cardiaque au repos : ________/min mesurée après plus d’une minute assis ou couché 0. pouls 80 ou moins 1. pouls entre 81 et 100 2. pouls entre 101 et 120 4. pouls à plus de 120
Sweating	Sweating: *over past 1/2 h not accounted for by room temperature or patient activity.* 0 no report of chills or flushing 1 subjective report of chills or flushing 2 flushed or observable moistness on face 3 beads of sweat on brow or face 4 sweat streaming off face	Sudation : évaluée depuis les 30 dernières minutes, sans prendre en compte la sudation causée par la température de la pièce ou l’activité du patient 0 aucun frisson ou rougissement 1 se plaint de frissons ou de rougissement 2 rougissement ou sudation évidente au visage 3 sueurs qui perlent sur le visage 4 sueurs abondantes qui coulent du visage	Clear, simple or no comments (*n* = 20) Suggested adding the English word “flushing” which is frequently used (*n* = 1) Concerns about weather or health condition that could cause sudation (*n* = 2) Suggested observing moist hands and arms (*n* = 1) Suggested using the word “rougeurs” instead of “rougissement” (*n* = 1) Suggest some minor rewording of the sentence that did not change the meaning (*n* = 3) Suggested adding the word “diaphorèse” (*n* = 1)	2. Sudation : évaluée depuis les 30 dernières minutes, sans tenir compte de la sudation causée par la température de la pièce ou l’activité du patient. 0. aucun frisson ou rougissement/rougeur 1. se plaint de frissons ou rougissement 2. rougissement ou sudation évidente au visage 3. sueurs qui perlent sur le visage (diaphorèse) 4. sueurs abondantes qui coulent du visage
Restlessness	Restlessness *Observation during assessment* 0 able to sit still 1 reports difficulty sitting still, but is able to do so 3 frequent shifting or extraneous movements of legs/arms 5 unable to sit still for more than a few seconds	Agitation : observée durant l’évaluation 0 capable de rester assis 1 déclare avoir de la difficulté à rester assis, mais est capable de le faire 3 change fréquemment de position ou mouvements involontaires des jambes/bras 5 incapable de rester assis pendant plus de quelques secondes	Clear, simple or no comments (*n* = 25) In the hospital, some patients may be in other positions than sitting down (*n* = 1) Asked for clarifications about the word “agité” (*n* = 1) Suggested minor rewording “observez l’agitation” (*n* = 1)	3. Agitation : observée durant l’évaluation 0. capable de rester assis 1. déclare avoir de la difficulté à rester assis, mais est capable de le faire 3. change fréquemment de position ou mouvements involontaires des jambes/bras 5. incapable de rester assis pendant plus de quelques secondes
Pupil size	Pupil size 0 pupils pinned or normal size for room light 1 pupils possibly larger than normal for room light 2 pupils moderately dilated 5 pupils so dilated that only the rim of the iris is visible	Taille des pupilles 0 grosseur normale à la lumière ambiante 1 possiblement plus grandes que la normale compte tenu de la lumière ambiante 2 dilatation modérée 5 dilatation sévère (l’iris n’est presque plus visible)	Clear, simple or no comments (*n* = 19) Suggested adding “importante” or “très importante” instead of “sévère” (*n* = 2) Expressed concern because pupil size is hard to measure due to proximity, lighting, tools to measure (*n* = 4) Suggested using millimeters (*n* = 2) Expressed concern about subjectivity (*n* = 1)	4. Tailles des pupilles 0. grosseur normale à la lumière ambiante 1. possiblement plus grandes que la normale compte tenu de la lumière ambiante 2. dilatation modérée 5. dilatation sévère ou importante/maximale (l’iris n’est presque plus visible)
Bone or joint aches	Bone or joint aches *if patient was having pain previously, only the additional component attributed to opiates withdrawal is scored* 0 not present 1 mild diffuse discomfort 2 patient reports severe diffuse aching of joints/muscles 4 patient is rubbing joints or muscles and is unable to sit still because of discomfort	Douleur osseuse ou articulaire Si le patient présentait de la douleur avant l’arrêt des opioïdes, ne tenir compte que des douleurs liées au sevrage 0 absent 1 inconfort diffuse 2 se plaint de douleur diffuse sévère des muscles/ articulations 4 patient se frotte les articulations/muscles et est incapable de rester tranquille à cause de la douleur	Clear, simple or no comments (*n* = 21) Great suggestion/agreed to separate previous pain and new onset of pain (*n* = 4) Should indicate “absente” instead of “absent” (*n* = 1) Indicated the description should be shorter (*n* = 1) Suggested using the word “immobile” or “rester en place” instead of “tranquille” (*n* = 2) Suggested removing “Si le patient… l’arrêt des opioïdes” (*n* = 1) Suggesting adding “comparé à son niveau de base” for the answer “absent” 0 (*n* = 1)	5. Douleur osseuse ou articulaire. Si le patient présentait de la douleur avant l’arrêt des opioïdes, ne tenir compte que des douleurs additionnelles liées au sevrage 0. absente 1. inconfort diffus 2. se plaint de douleur diffuse sévère des muscles/articulations 4. patient se frotte les articulations/muscles et est incapable de rester tranquille/assis ou en place à cause de la douleur
Runny nose or tearing	Runny nose or tearing *Not accounted for by cold symptoms or allergies* 0 not present 1 nasal stuffiness or unusually moist eyes 2 nose running or tearing 4 nose constantly running or tears streaming down cheeks	Rhinorrhée ou larmoiement : non causé par un rhume ou des allergies 0 absent 1 congestion nasale, yeux humides 2 rhinorrhée ou larmoiement 4 rhinorrhée constante ou larmes qui coulent sur les joues	Clear, simple or no comments (*n* = 22) Suggested to add « abnormalement » humide (*n* = 1) Suggested to add “et/ou” instead of “ou” (*n* = 1) Suggested to add “at the time of the interview” (*n* = 1) Suggested to reword “en absence de rhume ou d’allergies » (*n* = 1)	6. Rhinorrhée ou larmoiement : non causé par le rhume ou les allergies 0. absent 1. congestion nasale ou yeux anormalement humides 2. rhinorrhée ou larmoiement 4. rhinorrhée constante ou larmes qui coulent sur les joues
GI Upset	GI Upset: *over last 1/2 h* 0 no GI symptoms 1 stomach cramps 2 nausea or loose stool 3 vomiting or diarrhea 5 multiple episodes of diarrhea or vomiting	Inconfort gastro-intestinal : depuis les 30 dernières minutes 0 aucun symptôme gastro-intestinal 1 crampes abdominales 2 nausée ou selles molles 3 vomissements ou diarrhée 5 plusieurs épisodes de vomissements ou diarrhée	Clear, simple or no comments (*n* = 24) Concern about the short duration of observation (*n* = 2) Suggested to evaluate vomiting and diarrhea separately (*n* = 1) Suggested minor rewording (*n* = 2)	7. Inconfort gastro-intestinal : depuis les 30 dernières minutes 0. aucun symptôme gastro-intestinal 1. crampes abdominales 2. nausée ou selles molles 3. vomissements ou diarrhée 5. plusieurs épisodes de vomissements ou diarrhée
Tremor	Tremor: *observation of outstretched hands* 0 no tremor 1 tremor can be felt, but not observed 2 slight tremor observable 4 gross tremor or muscle twitching	Tremblements : observés avec les bras étendus, doigts écartés 0 aucun tremblement 1 tremblements sont ressentis, mais non visibles 2 tremblements légers 4 tremblements sévères ou spasmes musculaires	Clear, simple or no comments (*n* = 17) Suggested adding a « modérés » category between “légers” and “sévères” (*n* = 2) Suggested clarifying who feels the tremor (*n* = 5) Suggested changing “étendus” for “tendus” (*n* = 2) Suggested adding “tremblements observables” (*n* = 1) Suggested removing “observés avec les” (*n* = 1)	8. Tremblements : observer avec les bras étendus, doigts écartés 0. absent 1. tremblements sont ressentis, mais non visibles 2. tremblements légers 4. tremblements sévères ou spasmes musculaires
Yawning	Yawning: *observation during assessment* 0 no yawning l yawning once or twice during assessment 2 yawning three or more times during assessment 4 yawning several times/minute	Bâillements : observés durant l’évaluation 0 aucun bâillement 1 1–2 bâillements durant l’évaluation 2 3 bâillements ou plus durant l’évaluation 4 plusieurs bâillements/minute	Clear, simple or no comments (*n* = 27) Suggested replacing “observés” by “évaluation” (*n* = 1)	9. Bâillements : observer durant l’évaluation 0. aucun bâillement 1. 1–2 bâillements durant l’évaluation 2. 3 bâillements ou plus durant l’évaluation 4. plusieurs bâillements/minute
Anxiety or Irritability	Anxiety or Irritability 0 none 1 patient reports increasing irritability or anxiousness 2 patient obviously irritable or anxious 4 patient so irritable or anxious that participation in the assessment is difficult	Anxiété ou irritabilité 0 aucune anxiété ou irritabilité 1 se plaint d’anxiété ou d’irritabilité 2 anxiété ou irritabilité objectivable 4 patient si anxieux ou irritable que sa participation à l’évaluation est difficile	Clear, simple or no comments (*n* = 22) Suggested changing “objectivable” by “évidente” (*n* = 1) Option 4 is not clear and should be reworded (*n* = 3) Commented that it is difficult to evaluate/quantify anxiety (*n* = 1) Suggested adding consideration for baseline anxiety of the patient (*n* = 1)	10. Anxiété ou irritabilité 0. aucune anxiété ou irritabilité 1. se plaint d’anxiété ou d’irritabilité 2. anxiété ou irritabilité objectivable/évidente 4. patient si anxieux ou irritable que sa participation à l’évaluation est difficile
Gooseflesh skin	Gooseflesh skin 0 skin is smooth 3 piloerrection of skin can be felt or hairs standing up on arms 5 prominent piloerrection	Chair de poule 0 peau lisse 3 piloérection est ressentie, on voit les poils des bras redressés 5 piloérection importante	Clear, simple or no comments (*n* = 26) Suggested adding “piloerection” to the title, since “chair de poule” is not a medical term (*n* = 1) Suggested a minor rewording (*n* = 1)	11. Chair de poule ou piloérection 0. peau lisse 3. piloérection est ressentie, on voit les poils des bras redressés 5. piloérection proéminente
Total score	The total score is the sum of all 11 items. Total score: __________ Score: 5- 1 2 = mild; 1 3–24 = moderate; 25–36 = moderately severe; more than 36 = severe withdrawal	Voici l’évaluation du score obtenu au questionnaire : Score total : __________ Le score total est la somme de tous les 11 items Score : 5–12 = léger; 13–24 = modéré; 25–36 = modérément sévère; plus de 36 = sevrage sévère.	Clear, simple or no comments (*n* = 17) Useful (*n* = 2) Could indicate “severage” at each level (*n* = 3) Suggested rewording using “Interprétation: Severage d’intensité …” (*n* = 1) The score 0–5 is not present (*n* = 1) Could be useful to add an item for sleepiness, depending on where the patient is seen (*n* = 1) Asked clarification about the consideration of resting pulse rate if a patient exercised before the assessment (*n* = 1) Reword “somme de tous les items” (*n* = 1) Suggestion to improve the visual presentation (*n* = 1) Suggested adding a field for date & time of the assessment as well as a section to indicate time of last adjuvant medication intake (*n* = 1)	Score total : ___________ (Le score total est la somme de tous les items) Score de sevrage : 5–12 = Faible; 13–24 = Modéré; 25–36 = Modérément sévère; >36 = sévère

COWS: Clinical Opiate Withdrawal Scale.

## Results

All the steps (i.e., Steps 1–6) involved in the French-Canadian translation and cultural adaptation of the COWS took place between June and November 2019. Descriptive statistics for the sample of healthcare professionals who participated in the pretest of the COWS-FC are presented in Table 1. The majority of participants were women (78%), aged between 35 and 44 years (41%), and Caucasians (93%). Among these participants, nursing (52%) and medicine (37%) were the most frequent healthcare disciplines, and close to 90% of participants reported having worked with opioid users for more than 5 years.

Table 2 presents an overview of the results from the pretest (i.e., Step 5) conducted among healthcare professionals. The minor adjustments that were made to yield the final French-Canadian version of the COWS (i.e., COWS-FC) are also described in Table 2. Results from the pretest indicated that the vast majority of items from the prefinal version of the COWS-FC were clear and well understood by participants (i.e., healthcare professionals). However, based on participants’ suggestions, minor adjustments to certain words or statements were made to enhance clarity. For instance, this occurred for item # 4 (adding the words “*rougeurs*” and “*diaphorèse*”), item # 10 (adding the words “*assis en place*”) and item # 22 (adding the word “*piloérection*”). The wording of item # 12 in the final version was also slightly modified to more accurately represent one of the response choices (i.e., by adding the word “*anormalement*”). For the “total score” section, three participants suggested adding the word “*sevrage*” to the scoring legend, which was done in the final version to enhance clarity.

Some participants (*n* = 2) suggested adding one response choice (i.e., “*modérés*”) to item # 16, but this suggestion was not retained because it would have altered the scoring system implemented in the original version of the COWS. Similarly, suggestions were made to add additional items (e.g., an item assessing sleep disturbance and an item assessing the date/time of last adjuvant medication intake), but these suggestions were not retained because these items were not included in the original version of the COWS and this would have altered the original scoring system.

## Discussion

The main objective of the present study was to develop a French-Canadian translation and adaptation of the COWS (i.e., the COWS-FC) for the assessment of opioid withdrawal symptoms in clinical and research settings. A number of instruments have previously been developed and validated for the assessment of opioid withdrawal symptoms, but none have been culturally adapted for French-Canadian populations. Opioid withdrawal symptoms are frequently assessed both in clinical and research settings, and there was a need to develop a culturally adapted version of the COWS that could be used by French Canadians.

Because of its psychometric properties, ease of application and clinical utility, the COWS has become widely used for the assessment of opioid withdrawal symptoms among various populations of opioid users.^47–52^ Results from the pretest conducted among healthcare professionals with experience working with opioid users showed that the French-Canadian version of the COWS was generally well understood by participants, both in terms of general instructions and item wording. Given the very few adjustments that needed to be made following the pretest, it is unlikely that the clarity and understandability of items from the COWS-FC meaningfully varied as a function of healthcare professionals’ demographic or occupational characteristics. Finally, the pretest did not reveal any specific challenge that would need to be addressed. Overall, the pretest led only to minor modifications to the wording of certain items to enhance clarity, without any alterations to the meaning of items included in the original (i.e., English) version of the COWS. The comparability and absence of any meaningful differences between the original English and French-Canadian versions of the COWS is expected to be useful not only for clinical purposes but also to facilitate comparisons across studies involving the assessment of opioid withdrawal symptoms using the COWS.

## Strengths and Limitations

Rigorous steps based on the guidelines for the translation and cultural adaptation of assessment tools were followed, which led to a semantically equivalent version of the COWS. However, future studies that will use the COWS-FC should report its psychometric properties and compare them with those from the original version. Second, data from the pretest were collected among healthcare professionals from various disciplines having worked with opioid users for several years, but the distinction between healthcare professionals’ experience working with medical or nonmedical users was not made. It is unlikely that this distinction would have impacted healthcare professionals’ understandability of the COWS-FC, but this needs to be considered when interpreting results from the pretest. Finally, there are limitations associated with the initial version of the COWS that need to be considered. For instance, although the COWS has been used in clinical and research settings among patients prescribed opioids,^47,61,63–65^ it was primarily developed and validated for illicit opioid users. Further studies are needed to test the psychometric properties of the COWS among patients using opioids for medical reasons.

Despite these limitations, this study led to a linguistically and culturally adapted version of the COWS that is expected to be useful for the assessment of opioid withdrawal symptoms by French-Canadian populations. The linguistic and cultural adaptation conducted in the present study has broad applicability given that the COWS-FC is frequently used in both clinical and research settings. Clinicians and researchers working in francophone settings across Canada should now consider using the COWS-FC for the assessment of opioid withdrawal symptoms.

## Supplemental Material

sj-docx-1-cpa-10.1177_07067437221087066 - Supplemental material for French-Canadian Translation and Cultural Adaptation of the Clinical Opiate Withdrawal Scale: The COWS-FC Click here for additional data file. Supplemental material, sj-docx-1-cpa-10.1177_07067437221087066 for French-Canadian Translation and Cultural Adaptation of the Clinical Opiate Withdrawal Scale: The COWS-FC by Alice Bruneau, Clarice Poirier, Mélanie Bérubé, Aline Boulanger, Céline Gélinas, Line Guénette, Anaïs Lacasse, David Lussier, Yannick Tousignant-Laflamme, M. Gabrielle Pagé, Marc O. Martel and in The Canadian Journal of Psychiatry
