# Supplementary material for: French-Canadian Translation and Cultural Adaptation of the Clinical Opiate Withdrawal Scale: The COWS-FC
Source: Can J Psychiatry. 2022 Mar 15;67(9):701–11. doi: 10.1177/07067437221087066 (PMC9449138; doi:10.1177/07067437221087066)
Supplement: sj-docx-1-cpa-10.1177_07067437221087066 - Supplemental material for French-Canadian Translation and Cultural Adaptation of the Clinical Opiate Withdrawal Scale: The COWS-FC [file sj-docx-1-cpa-10.1177_07067437221087066.docx]

**ÉCHELLE DU SEVRAGE CLINIQUE DES OPIOÏDES**
(COWS-FC : CLINICAL OPIATE WITHDRAWAL SCALE–FRENCH CANADIAN)


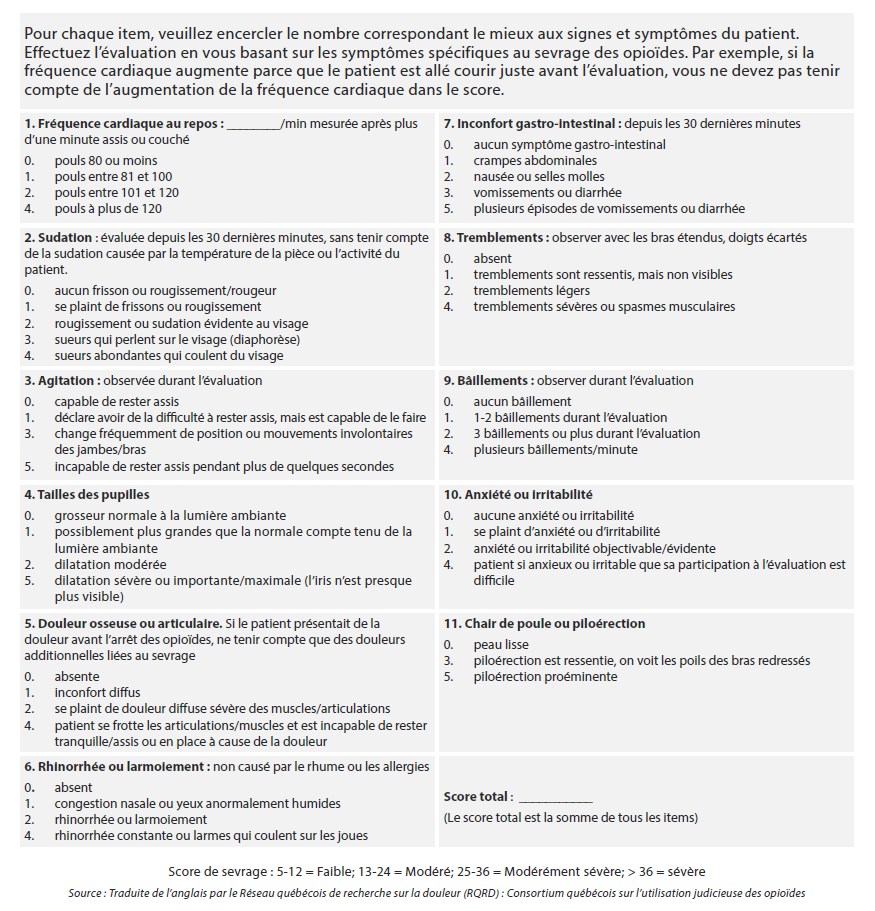


*Cette version peut être copiée et utilisée*
